# Gastrostomy Exchange With Displacement Into the Jejunum

**DOI:** 10.7759/cureus.57444

**Published:** 2024-04-02

**Authors:** Daniel I Mazzorana, Kamil Arif, Junjian Huang

**Affiliations:** 1 Medical School, Alabama College of Osteopathic Medicine, Dothan, USA; 2 Interventional Radiology, University of Alabama at Birmingham, Birmingham, USA

**Keywords:** intussusception, gastrostomy, gastroenteric, gastroduodenojejunal, gastrostomy tube, fundus

## Abstract

A 54-year-old female with a gastrostomy tube presented with persistent nausea, vomiting, and abdominal pain. On physical examination, the gastrostomy tube was completely advanced into the abdominal cavity with only the external retention ring and hub on the exterior. The first step in the workup was obtaining a scout radiograph. This imaging was appropriate to show the location of the gastrostomy catheter which was overlying the left upper quadrant. The distal tip was heading in the direction of the left lower quadrant. The course of the gastrostomy catheter did not follow the expected direction of the duodenal C-loop. Proceeding forward, contrast was injected through the existing catheter which showed opacification of the jejunal bowel loops. The patient could now be properly diagnosed with gastroenteric intussusception and proper measures could be taken. Following the injected contrast showing jejunal opacification, a stiff Glidewire was advanced through the existing catheter all the way into the jejunum. Deflation of the balloon and removal of the catheter caused an almost instantaneous change in the course of the Glidewire to the more conventional path following the course of the duodenal C-loop. A sheath was then advanced over the wire which was now shown crossing the midline in the proper gastroduodenojejunal course. The final step was to advance a new 22-French MIC gastrostomy catheter over the Glidewire. The contrast was then injected to demonstrate proper opacification of the rugal folds and gastric fundus. The patient reported an immediate resolution of symptoms and was later discharged.

## Introduction

Individuals unable to eat or who have trouble eating require the use of a gastrostomy tube to meet nutritional needs. This necessity may arise from a spectrum of underlying health conditions ranging from Crohn’s Disease, where the inflammatory nature of the condition can impair normal digestive processes, to neurological ailments such as amyotrophic lateral sclerosis (ALS), which causes a gradual weakening of muscles involved in swallowing and feeding [1]. Recently, radiology has assumed an increasingly pivotal role in the placement and management of gastrostomy tubes. Through techniques such as fluoroscopy and ultrasound guidance, radiologists can ensure that the catheters are placed precisely which minimizes the risk of complications while optimizing patient outcomes. Furthermore, imaging modalities make it easier to monitor the tube positioning and possibly detect any potential issues post-procedure which allows for timely intervention if necessary. Although these procedures are regarded as being relatively safe, up to 5% of patients will have postoperative complications [2]. Complications of these procedures may include, but are not limited to, gastric perforation, peritonitis, gastric bleeding, yet rarely gastric volvulus or intussusception [3]. There are instances in which there may be inflammation at the site of the gastrostomy tube and possible displacement due to traction or abnormal movement.

## Case presentation

A 54-year-old female patient presented with a constellation of symptoms, including persistent nausea, vomiting, and diffuse abdominal pain. These symptoms, coupled with her medical history, raised a significant concern regarding her gastrointestinal health. Of note was her history of ALS which had led to muscle weakness and atrophy. In October 2021, the patient underwent a routine gastrostomy tube placement due to intolerance of oral feeding which is a common issue in ALS patients. As the disease progresses, it can affect swallowing function. This procedure involves inserting a tube directly into the stomach percutaneously through the abdominal wall. The goal is to provide the patient with nutrition and medications. The pivotal event in this case appeared to be the adjustment of the gastrostomy tube catheter by a home health nurse. This caused the patient discomfort in the coming hours. The adjustment likely perturbed the positioning of the catheter and led to its migration entirely into the jejunum. During the physical examination, the only thing that was present on inspection was the external retention ring and the hub. This inadvertent migration of the gastrostomy tube catheter raised several immediate concerns. The functionality could potentially be compromised and potentially worsen the patient’s condition. The mechanical discomfort was likely exacerbating ALS symptoms and increasing the risk of complications, necessitating urgent intervention such as repositioning or ultimately a complete replacement. In summary, the patient’s presentation of persistent nausea, vomiting, and diffuse abdominal pain, in the context of ALS and a recent gastrostomy tube replacement, underscores the importance of careful monitoring and management of medical devices in vulnerable patient populations. Timely recognition and intervention are crucial to mitigate complications and optimize patient outcomes.

**Figure 1:**
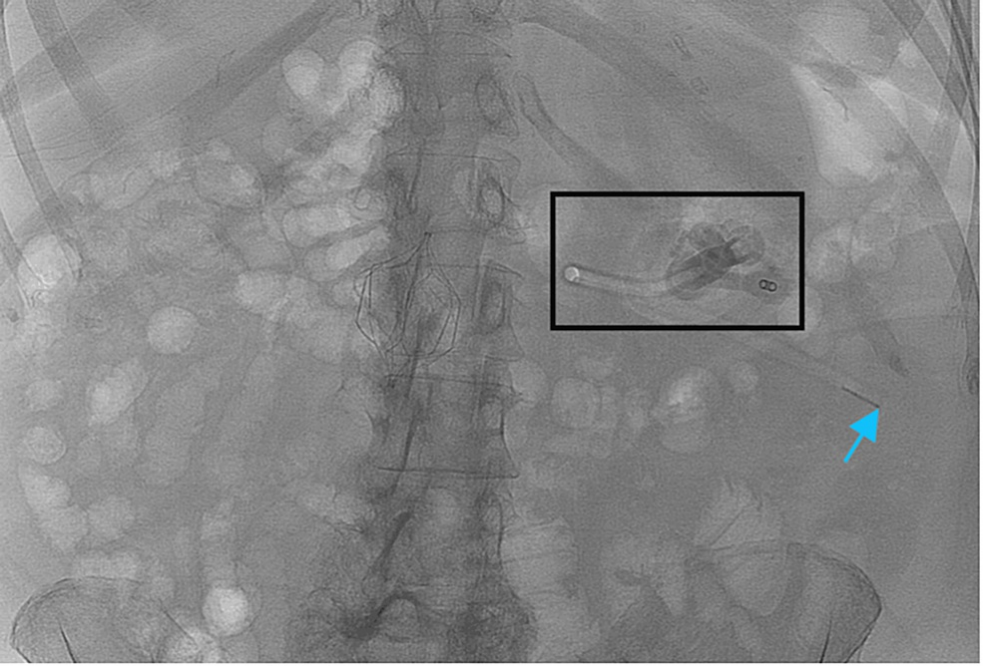
Scout radiograph demonstrates the gastrostomy catheter overlying the left upper quadrant (black box) with the distal tip coursing toward the left lower quadrant (blue arrow).

**Figure 2:**
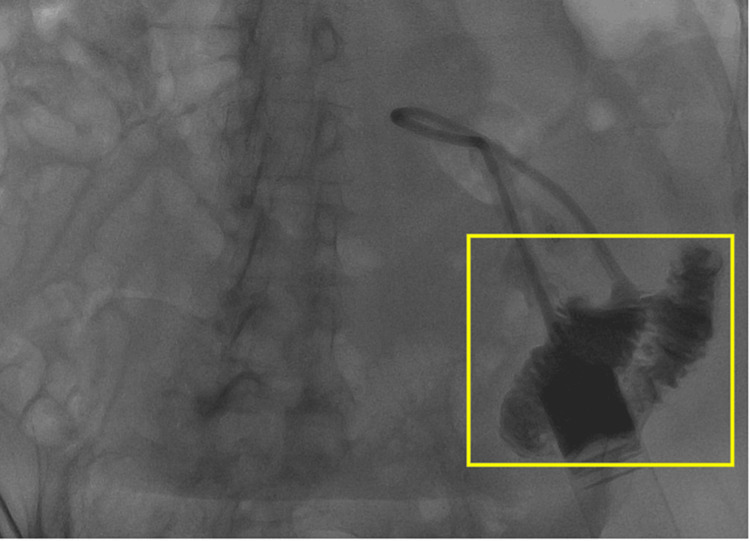
Contrast injected through the existing catheter demonstrates opacification of jejunal bowel loops (yellow box).

**Figure 3:**
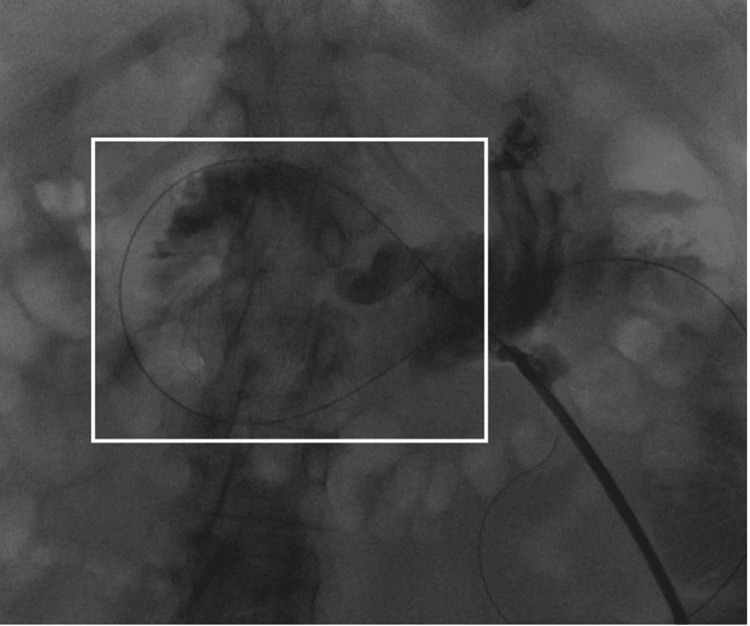
A stiff Glidewire was advanced through the existing catheter into the jejunum. The balloon was deflated, the catheter removed, and a sheath placed over the wire. Following the removal of the gastrostomy catheter, there was an immediate change in the configuration of the wire course. The wire now crossing the midline and outlining the expected gastroduodenojejunal course (white box).

**Figure 4:**
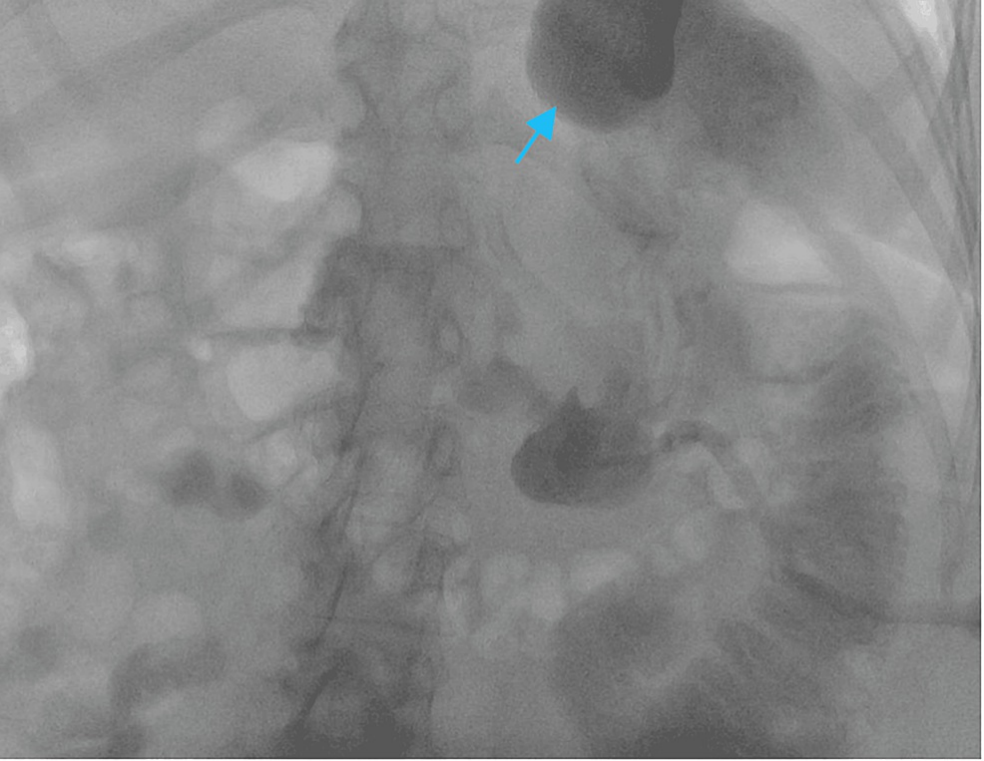
A new 22-French MIC gastrostomy catheter was advanced into the stomach over the wire. Contrast was injected with opacification of rugal folds and gastric fundus (blue arrow).

Given the course of catheter tubing on the scout radiograph shown in Figure 1 and the initial lack of duodenal opacification in Figure 2, a gastrojejunal fistula was considered in the initial differential. Removing the existing malpositioned gastrostomy catheter and performing a sheath pull-back confirmed that this was not the case, however. Another complication that presents similarly is a gastric volvulus which has also rarely been associated with gastrostomy malposition. Nevertheless, with a gastric volvulus, there is preservation of the duodenal course. Therefore, the expected course of the gastrostomy catheter tubing should have crossed the patient’s midline and followed the usual “C” shape of the duodenum. The correct course is demonstrated in Figure 3 where the removal of the gastrostomy catheter led to the resolution of symptoms and now the correct configuration of the wire course. After ruling out other possibilities, the final diagnosis that was reached was gastroenteric intussusception. Inadvertent catheter and retention balloon malposition into the jejunum resulted in a partially obstructive gastric intussusception. The gastrostomy balloon was the lead point for the gastroenteric intussusception. Again, the common clinical presentation includes nausea, vomiting, and abdominal pain. Once the new catheter was positioned, contrast was injected to confirm that it was in the proper position, as shown in Figure 4.

## Discussion

Previous cases of gastroenteric intussusception provide valuable insights into the clinical presentation, diagnostic challenges, and different management strategies for this condition. Gastroenteric intussusception is a rare occurrence where a patient’s stomach is folded into the subsequent intestinal section. Reviewing similar cases can help clinicians recognize patterns and refine their approach for proper diagnosis and treatment plans.

For example, a case study highlighted the rarity of this condition with an incidence rate of only 0.15%. Despite its low frequency, the study demonstrated the importance of keeping in mind gastroenteric intussusception in patients presenting with nausea, vomiting, and abdominal pain. Furthermore, this case underscored the need for a prompt diagnosis and intervention to prevent any potential complications [4]. Similarly, a systematic review shed light on the clinical presentation and management in patients who have undergone gastric bypass surgery. This study identified symptoms such as nausea, vomiting, and abdominal pain as common symptoms of gastric intussusception post-gastric bypass. The review emphasized the importance of clinical suspicion and thorough examination in cases such as these [5].

This triad of symptoms can be considered a vague presentation with a long list of differential diagnoses. It is imperative to perform a comprehensive physical examination to narrow the scope of differentials. In a case such as this one, it is important to examine the patient and demonstrate abnormal positioning of the catheter tubing or retention bumper.

Gastric intussusception can be reliably identified on cross-sectional imaging or fluoroscopic studies. Administering contrast to the upper gastrointestinal region is done to evaluate the anatomy and the passage of contrast. This allows differential diagnoses to be ruled in or out due to the associated obstructions. Most commonly, an upper gastrointestinal study is performed to show the esophagus, stomach, and duodenum. On CT imaging, gastric intussusception is typically represented as a target-like mass. The concentric bowel wall thickening causes this characteristic layering effect [6]. Furthermore, the lead point can be found most distal to the intussusception. When observing the catheter in suspected gastroenteric intussusception, it will not cross the midline. There is also a lack of opacification of the expected gastric rugal folds when injecting contrast through the existing catheter. This study faces limitations due to the scarcity of comparable cases, hindering direct comparison and statistical analysis, without a robust dataset, evaluating trends or patterns becomes a challenge. However, its strength lies in providing valuable reference material for clinicians encountering similar presentations, offering insights that can guide diagnosis and treatment strategies. By documenting and sharing unique cases, this study contributes to the collective knowledge base, potentially informing future clinical decisions and improving patient care.

## Conclusions

When placing or manipulating a gastrostomy tube, ensuring the proper position of the gastrostomy tube is fundamental to the procedure. In cases where this is not successfully accomplished, it can lead to gastrostomy tube migration, rarely causing intussusception. Usually, adjusting the catheter to the appropriate position or removing the catheter will lead to a resolution of the intussusception. If the issue is not resolved by removing the existing catheter promptly, surgery may be indicated with resection of the affected bowel segment.
